# Enrichment analysis of phenotypic data for drug repurposing in rare diseases

**DOI:** 10.3389/fphar.2023.1128562

**Published:** 2023-07-25

**Authors:** Alberto Ambesi-Impiombato, Kimberly Cox, Sylvie Ramboz, Daniela Brunner, Mukesh Bansal, Emer Leahy

**Affiliations:** PsychoGenics, Paramus, NJ, United States

**Keywords:** drug discovery, phenotypic screening, tianeptine, huntington (disease), animal models, smartcube, computational method

## Abstract

Drug-induced Behavioral Signature Analysis (DBSA), is a machine learning (ML) method for *in silico* screening of compounds, inspired by analytical methods quantifying gene enrichment in genomic analyses. When applied to behavioral data it can identify drugs that can potentially reverse *in vivo* behavioral symptoms in animal models of human disease and suggest new hypotheses for drug discovery and repurposing. We present a proof-of-concept study aiming to assess Drug-induced Behavioral Signature Analysis (DBSA) as a systematic approach for drug discovery for rare disorders. We applied Drug-induced Behavioral Signature Analysis to high-content behavioral data obtained with SmartCube^®^, an automated *in vivo* phenotyping platform. The therapeutic potential of several dozen approved drugs was assessed for phenotypic reversal of the behavioral profile of a Huntington’s Disease (HD) murine model, the Q175 heterozygous knock-in mice. The *in silico* Drug-induced Behavioral Signature Analysis predictions were enriched for drugs known to be effective in the symptomatic treatment of Huntington’s Disease, including bupropion, modafinil, methylphenidate, and several SSRIs, as well as the atypical antidepressant tianeptine. To validate the method, we tested acute and chronic effects of tianeptine (20 mg/kg*, i. p.*) *in vivo*, using Q175 mice and wild type controls. In both experiments, tianeptine significantly rescued the behavioral phenotype assessed with the SmartCube^®^ platform. Our target-agnostic method thus showed promise for identification of symptomatic relief treatments for rare disorders, providing an alternative method for hypothesis generation and drug discovery for disorders with huge disease burden and unmet medical needs.

## Introduction

Applying advanced analytical methods and machine learning to biological data has great promise for drug discovery and development. An area that would benefit greatly is the repurposing of drugs for rare disorders that lack effective therapeutic treatments. Partly due to the lack of understanding of the key biological underpinnings driving disease presentation and progression, drug discovery for rare disorders lags behind other drug discovery efforts. With this in mind, we developed a method to repurpose approved drugs (or, alternatively, identify novel treatments) for behavioral symptom relief: Drug-induced Behavioral Signature Analysis (DBSA).

### From gene expression to behavior

DBSA was inspired by gene expression enrichment analysis (GSEA), used in the genomic and bioinformatic areas of research, to identify genes or pathways significantly affected by a disease (Subramanian et al., 2005). GSEA has been used to identify drugs or compounds based on their potential to reverse the disease signatures (Lamb et al., 2006). Similarly, DBSA defines behavioral symptoms that are exacerbated in an animal model of disease and finds putative treatments with the potential to normalize such disease-driven phenotypes. The application to behavioral data, involving the identification of behavioral symptoms affected by a disease, requires both a good animal model of the target disease, and a large dataset of drug phenotypic signatures using the same species. Hypotheses regarding possible treatments can then be followed by direct *in vivo* testing of the novel ideas in the target animal model, as a first-tier validation.

**FIGURE 1:**
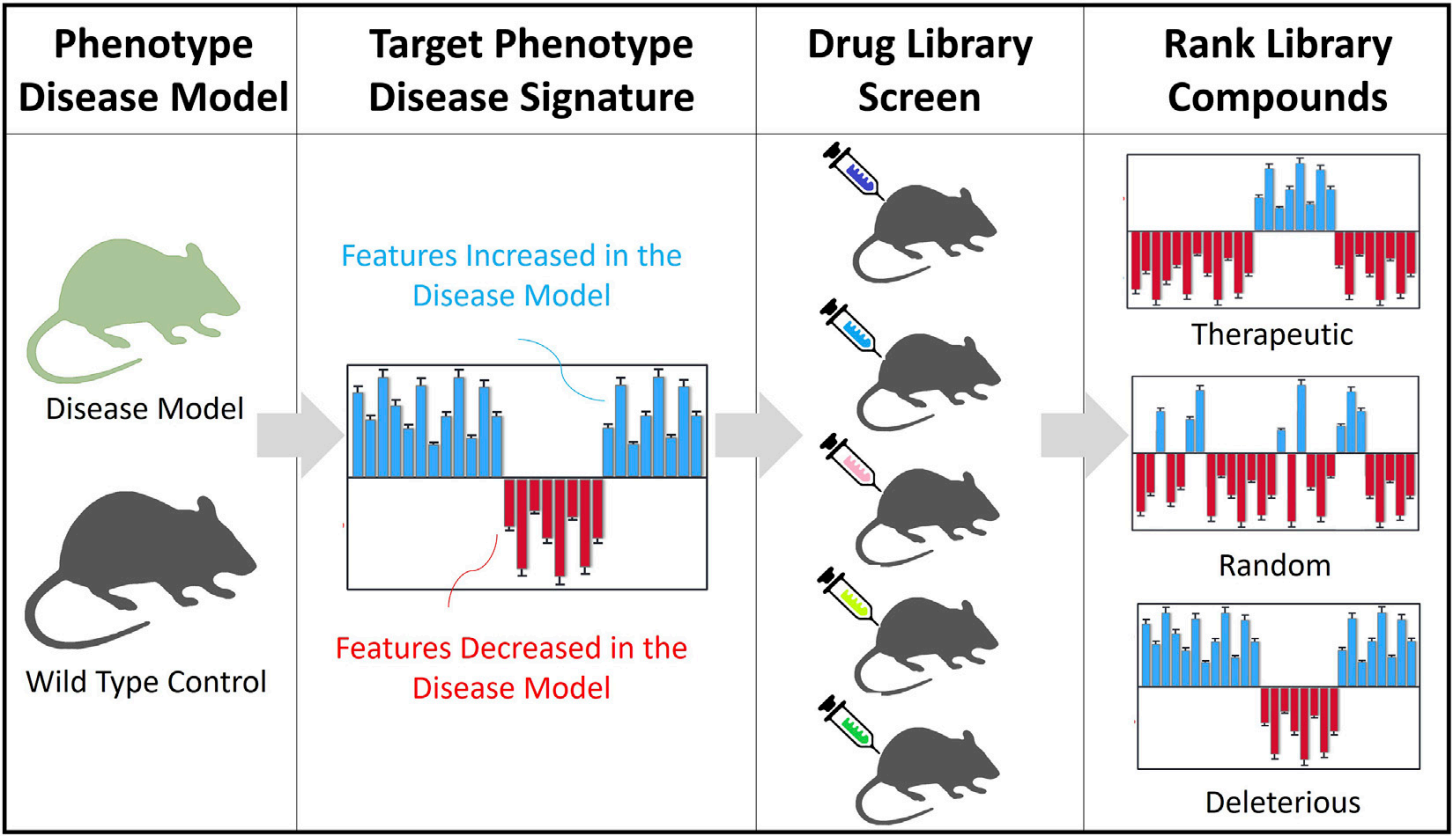
Overview of the DBSA method. *Target Phenotype of the Disease Model*: Animal models of a rare disease and their wild type controls are tested through a high throughput standardized behavioral phenotyping platform. *Target Phenotype Disease Signature:* Identify top features and the direction of the change. A theoretical example shows increased (blue) and decreased (red) features, relative to WT controls. *Drug Library Screen:* Testing *in vivo*, in WT mice, compounds are assessed for their phenotypic effects on behavior (using the same standardized phenotyping platform). Behavioral feature profiles are generated for the WT mouse groups treated with each compound, comparing their effects with a vehicle group (increased in blue, decreased in red). *Ranking of Library Compounds*. The compound profiles are analyzed against the target disease signature to rank compounds according to their potential to reverse or worsen the disease phenotype.

This study explores the utility of DBSA, using phenotypic behavioral drug effect profiles, compared against phenotypic data of a *target* animal model of Huntington’s Disease (HD). The target behavioral signature derived from an animal model consists of increased or decreased behavioral features relative to the *wild typ*e (WT) control (Figure 1). This *target* signature is then systematically compared to drug signatures from the library, consisting of drug-induced effects on the full set of measured behavioral features relative to vehicle-treated controls, in WT mice. Compounds with robust significant reversal of the model signature are identified as those with the greatest potential for repurposing. Candidate compounds can be further validated in follow-up studies where the target model is treated to test whether the phenotype is rescued.

### Behavioral data

A requirement for the application of DBSA to *in vivo* phenotypic data, is that the behavioral assessment should be done in a standardized manner and should be comprehensive. Standardization ensures that drug datasets can be compared to animal model datasets, whereas the comprehensive scope of the phenotype assessment facilitates translation to the clinic. To satisfy both requirements we collected behavioral data with the SmartCube^®^ platform using a standard protocol to characterize *in vivo* drug signatures of the HD model relative to their WT controls, as well as the library compounds relative to their vehicle controls. We finally tested the top drug candidate resulting from the screening, again using the same standard protocol, after administering the drug candidate to the HD model mice. This platform employs computer vision and mechanical actuators to detect spontaneous and evoked behavior eliciting responses through anxiogenic, startling, and other stimuli. Behavioral readouts include locomotion, trajectory complexity, body posture and shape, simple behaviors, behavioral sequences, and other features describing minute body shape and movement (Roberds et al., 2011; Alexandrov et al., 2016; Kabitzke et al., 2020). These key features enable use of drug signatures from several dozen drugs at many doses (see Supplementary Table S1), which have been collected using the same protocol (including mouse strain, vehicle, and administration route).

### Huntington’s disease (HD)

Another key aspect of the method is the choice of a suitable animal model of the targeted disease. In this study, we applied DBSA to identify novel therapeutics for HD, a devastating rare neurodegenerative disorder with a prevalence of 5–7 cases per 100,000 (Walker, 2007). HD is caused by the autosomal dominant inheritance of a CAG trinucleotide repeat expansion within the huntingtin gene (*HTT*). Expanded CAG repeat results in an abnormal form of the huntingtin protein (mHtt) affecting the polyglutamine region of the protein. While the pathogenesis of HD is well described and its genetic causes were established decades ago, there are still no available cures, and its progressive nature results in a huge unmet medical need.

### Animal model of HD

Many transgenic and knock-in (KI) mouse, rat, pig, and sheep models of HD exist, and several mouse models have been characterized using the SmartCube^®^ technology, making them particularly interesting for the present work. Behavioral features of HD models captured by this platform seem to correlate to, and are predictive of, the underlying pathology, as machine learning models trained on behavioral data can be used accurately predict the size of the CAG expansion (Alexandrov et al., 2016). Longer CAG repeats result in both early manifestation and increased severity of symptoms, mimicking the progression of HD, despite important differences between the mouse and human huntingtin mutations. We chose the heterozygous KI Q175 model, which has been extensively characterized (Menalled et al., 2012; Alexandrov et al., 2016). The Q175 models show changes in striatal genes expression starting around 3 months of age (Menalled et al., 2012), and behavioral changes soon thereafter with clear cognitive and motor deficits between 6 and 7 months of age, in both the original and a derived Q175 model (Curtin et al., 2016; Wu et al., 2022). Although cognitive aspects of HD are extremely important and can be studied in the lab (Farrar et al., 2014), the present work only focuses on motor function, exploratory behavior, response to aversive stimuli, and simple aspects of learning as adaptation to a novel environment, which can be captured in a high-throughput manner.

### Drug library screen

The drug library used for this work comprises 77 reference drugs, including antidepressants (e.g., bupropion, citalopram, fluoxetine, imipramine, tianeptine), antipsychotics (e.g., aripiprazole, chlorpromazine, clozapine, haloperidol, olanzapine), analgesics (e.g., acetylsalicylic acid, codeine, morphine, naproxen, oxycodone), anxiolytics (e.g., alprazolam, buspirone, chlordiazepoxide, MPEP, oxazepam), psychostimulants (e.g., D-amphetamine, caffeine, cocaine, methylphenidate), anticonvulsants/mood stabilizers (carbamazepine, gabapentin, lamotrigine, lithium, tiagabine, valproate), hallucinogens (DOI, MK801, phencyclidine), and other CNS drugs. We only considered drugs profiled using a wide dose range to characterize low and medium therapeutic effects, and to capture side effects at higher doses (Supplementary Table S1).

## Results

### The Q175 phenotype

Data from a previous large phenotyping study (Alexandrov et al., 2016) was used for the present report. Three cohorts of Q175 mice, screened at 2, 6, and 10 months of age, were compared with the corresponding WT mice to generate a phenotypic signature, and extract the top features that drive the phenotype at each age using the Decorrelated Ranked Feature Analysis (DRFA) (Alexandrov et al., 2016). This machine learning approach quantifies the phenotypic separation of two experimental groups (Q175 mice and WT control, in this case), in terms of a *Discrimination Index* (which measures the ability of a machine learning algorithm to correctly classify random subsets of the behavioral data). A useful feature of the DRFA analysis is that it also allows quantification of the phenotypic treatment *rescue* of the target model phenotype (*Recovery Index;* see Methods). DRFA is accompanied by 2D visualization of the groups’ statistical estimates (mean, standard error, and standard deviation) and their separation in the discriminant space, and a *p*-value calculation derived from repeated subsampling of the datasets (see Methods). Our screening platform could identify behavioral changes that are consistent with the expected HD phenotype in animal models and that can be broadly mapped to expected traditional behavioral domains.

**FIGURE 2:**
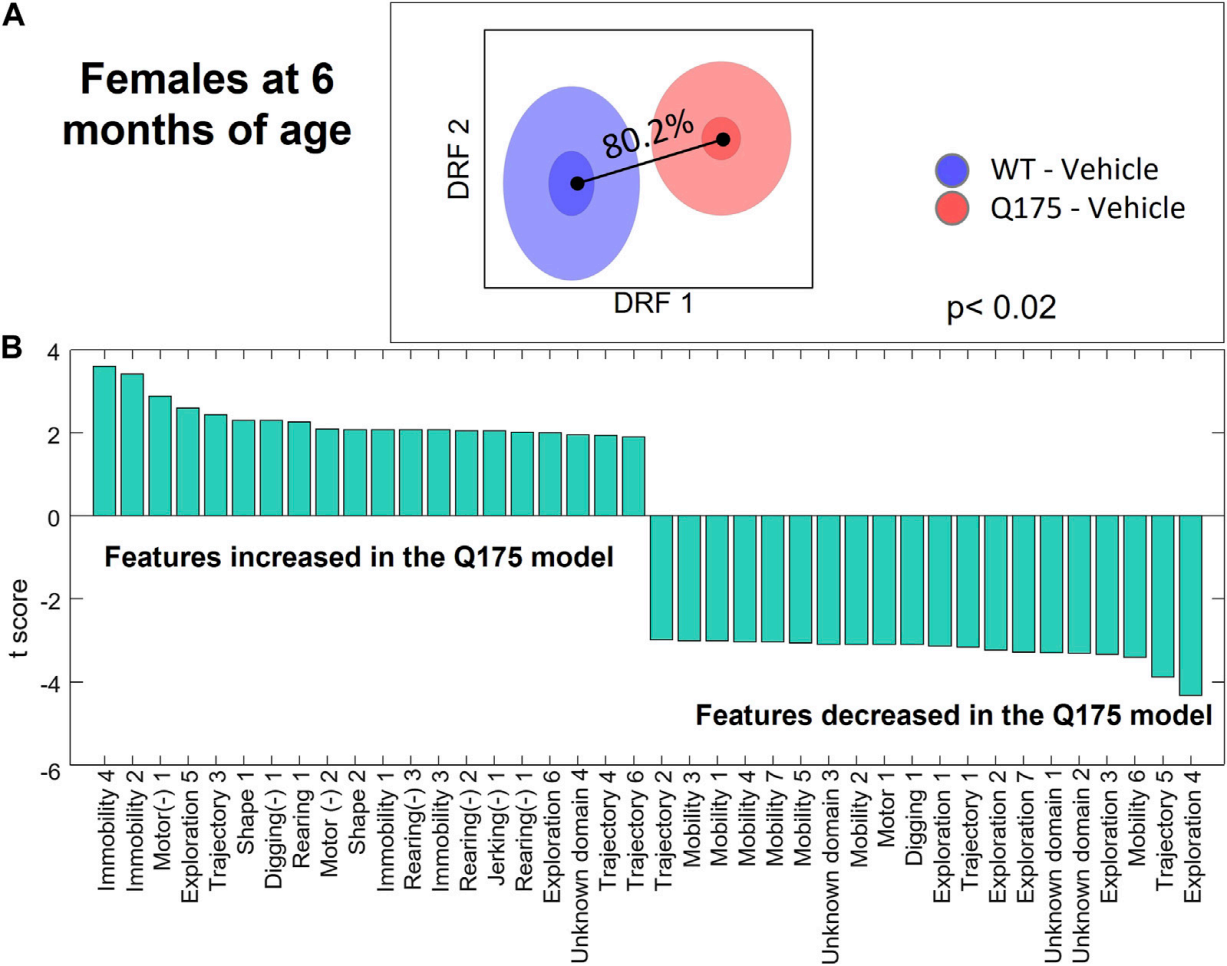
Differential behavioral features for female Q175 HD mice at 6 months. **(A)** Overall discrimination between HD and WT groups in the decorrelated feature space of DRFA, corresponding to a Discrimination Index of 80.2% (*p* < 0.02). **(B)** T-scores for the top features differentiating the two groups that are entered in the DBSA computational model. Left- and right-most features are those more robustly increased or decreased in the Q175 model as compared to the WT group. Features noted with the (−) symbol represent latencies. Numerical suffixes indicate variants of the same behavior, observed at different times in the experimental session.

The Discrimination Index for the Q175 model (Figure 2 and Supplementary Figure S1) showed a small difference at 2 months of age (Males: 63.7%, *p* < 0.08; Females: 67.2%, *p* < 0.03), which progressed to significant differences at 6 months (Males: 72.7%, *p* = 0.01; Females: 80.2%, *p* < 0.02) and 10 months of age (Males: 89.6%, *p* = 0.006; Females: 99.4%, *p* < 0.001). The top HD features that were consistently affected in Q175 mice relate to motor function and exploration (Figure 2 and Supplementary Figure S2). Specifically, exploration, locomotion, digging, and startle amplitude were decreased, and immobility and freezing were increased.

### DBSA screening of drug library

The 77 drugs screened in WT mice comprised a total of more than 200 doses (Supplementary Table S1). The different doses were independently ranked by their potential to reverse the HD phenotype, according to DBSA, and the top predictions were identified for each gender and each age group tested. To decrease the false positive rate, we considered only drugs predicted in both male and female. Table 1 shows the results for the top 8 drugs and their best doses, for males and females at different ages, separately. We considered the 6 months of age the most relevant age, as model mice show clear symptoms (as compared to the 2 months old subjects) but still present with good general health (in contrast to the 10-month-old mice). Sex-consistent results in the 6-month-old mice were modafinil, bupropion, methylphenidate and tianeptine.

**TABLE 1:** Summarized results of the DBSA screening: doses (mg/kg) of reference compounds predicted to reverse the HD phenotype in both males and female Q175, at any of the three different ages.

	Male			Female			
Age (months)	2	6	10	2	6	10	
Modafinil		30, 60, 120			30, 60, 120	15, 30, 60, 120	
Bupropion		8, 32			8	8, 16, 32	
Methylphenidate		5			5	2.5, 5, 10	
Escitalopram			60		12	3, 6, 12	
Tianeptine	60	20, 60			60		
Amphetamine		4	0.5, 2			4	
Caffeine					10	5, 10	
Chlordiazepoxide			24		4	4	

**FIGURE 3:**
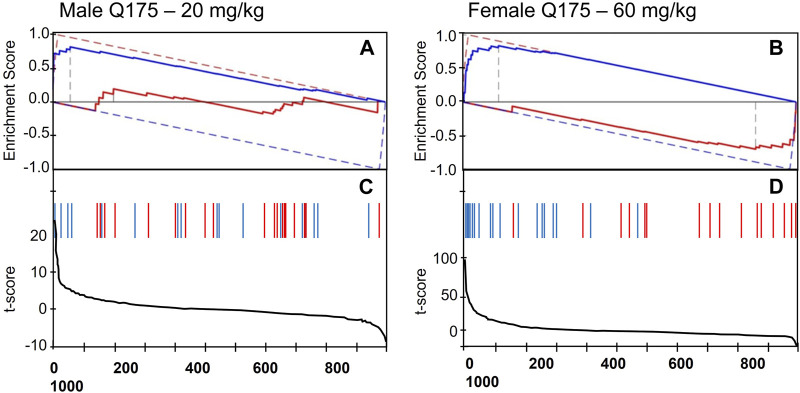
DBSA results. Enrichment Score showing reversal prediction of the phenotype of **(A)** 6-month-old male Q175 mice by 20 mg/kg of tianeptine, and **(B)** 6-month-old female Q175 mice by 60 mg/kg tianeptine. The red and blue solid lines represent the running enrichment scores for the increased and decreased features, respectively. The corresponding dashed lines present the theoretical maximum running enrichment scores. **(C,D)** Line graphs depicting the t-score for the full set of behavioral features when comparing Tianeptine treatment vs. vehicle in normal mice. Red and blue vertical bars mark the position of the features that were increased (blue) or decreased (red) in the Q175 mice as compared with the WT group.

Figure 3 depicts the detailed DBSA results for tianeptine, showing reversal of the phenotype of the 6-month-old male and female Q175 mice by tianeptine treatment (20 and 60 mg/kg, respectively). While tianeptine was predicted to reverse the HD phenotype in both males and females, the male mice showed a more robust effect, with reversal seen at both 2 months and 6 months with 60 mg/kg, and with the lower dosage of 20 mg/kg at 6 months. Female data suggested that at 6 months only the higher dose of 60 mg/kg would be potentially useful. At this dosage the DBSA predicted that reversal would be robust with many HD differential features showing opposite changes with tianeptine treatment (Figure 3D).

As the previous analysis consisted of *in silico* predictions of drug effects for the target HD model, and tianeptine was administered in normal mice only, we followed up with an *in vivo* study directly treating HD mice with tianeptine, to validate its therapeutic potential, and therefore the utility of DBSA for drug discovery.

### Tianeptine test in Q175 mice

To validate the prediction that tianeptine can reverse, to some extent, the phenotype of HD model mice, we tested tianeptine in an independent cohort of Q175 mice and WT controls of the same genetic background. As the DBSA predicted a relatively weaker response in female mice, with only the higher dose of 60 mg/kg predicted to be effective at 6 months, we hypothesized that a dose of 20 mg/kg would be able to ameliorate HD symptoms if given chronically. Female Q175 mice were therefore treated with daily *i. p.* injections, with either tianeptine (20 mg/kg) or vehicle for 4 weeks. Female WT controls were also chronically treated with vehicle. Male Q175 received one injection of tianeptine (20 mg/kg) or vehicle. Male WT controls were acutely treated with vehicle.

**FIGURE 4:**
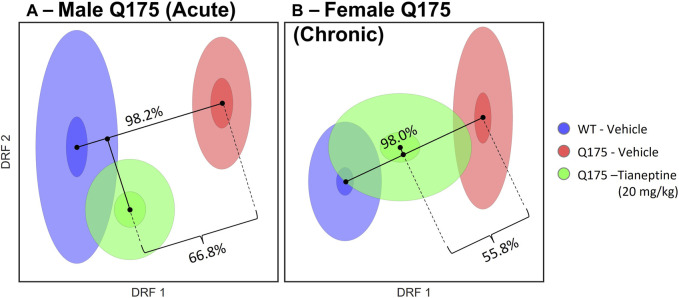
Effect of Tianeptine treatment on the Q175 HD model. Q175 mice were treated **(A)** acutely or **(B)** chronically with tianeptine (20 mg/kg) or saline. WT mice were treated with saline. A DRFA method was used to analyze all phenotypic data *in toto*. DRFA showed significant differences between Q175 and WT groups, as expected (98.2% and 98.0% for the males and females, respectively; ps < 0.0005). The tianeptine-treated group was used as a test set, to assess if they were more similar to the vehicle-treated Q175 group or to the WT controls. DRFA showed significant rescue of the Q175 phenotype by tianeptine (66.8% and 55.8% for males and females, respectively).

Analysis of behavioral results using the DRFA method showed, as expected, that male and female Q175 mice were significantly different from their corresponding WT control mice (with Discrimination Indices of 98%; *p*s< 0.001, Figure 4). Tianeptine significantly rescued the behavioral phenotype in both acute and chronic experiments, with 66.8% (*p* < 0.001) and 55.8% (*p* = 0.002) reduction of the disease phenotype, respectively (Figure 4). Results in the male Q175 group were tighter, with many of the predicted effects being realized (Supplementary Figure S3). Tianeptine mainly reversed decreased mobility and digging, and normalized rearing, in the Q175 male mice. Reversal in female Q175 was not so robust, although still significant, with effects on specific features not being as consistent as with the male group. The top features rescued by tianeptine in the Q175 female mice were decreased mobility and exploration.

## Discussion

### The DBSA method

The DBSA method is an *in silico* phenotypic screen of compound libraries that identifies those compounds with potential to reverse a behavioral phenotype for a particular disease. In contrast to a gene expression enrichment analysis, which may be a more direct assessment of the etiopathology of disease, behavioral enrichment analysis only assesses downstream behavioral manifestations in animal models of disease. DBSA is therefore based on the simple idea that a drug affecting behaviors in normal mice in the opposite direction than is observed in disease model animals, may have the potential to restore those behaviors if the model animals were treated with such drug. Many factors may help to disprove this hypothesis. First, drug effects in WT mice may engage different mechanisms of action than in animal models of disease. In the case presented here, however, tianeptine effects in WT mice closely resembled the effects in Q175 mice (Supplementary Figure S4). Second, disease-modifying and symptomatic treatment may require chronic treatment, not possible for a quick *in silico* screen. (Compound libraries in our system are administered acutely to healthy mice: chronic treatments will be prohibitively expensive.) Despite these valid concerns, DBSA is attractive because there are no quick and efficient ways to systematically generate hypothesis regarding novel mechanisms of action or drug repurposing for relief of disease burden. For most rare diseases, even for those disorders where the gene defect is known, no treatment exists due to either lack of knowledge or the complexity of the underlying biology. Small markets and reduced profitability results in low investment in R&D for these diseases. Thus, the ability to screen large libraries of test compounds in disease models for drug repurposing has been recognized as a game changer (Bellomo et al., 2017; Wu et al., 2022). Our method extends phenotypic drug-repurposing from cell assays and small animal (worms, flies, fish) screens to whole rodent *in silico* screen as a first step. In the context of large library screening, it is of sufficient value to rank potential compounds to generate hypotheses *in silico*. These hypotheses can then be assessed with regards to their mechanism of action, or, in a more agnostic manner, tested *in vivo* in the appropriate disease model.

### Drug library

The compound library used in this study consisted of a large compendium of reference CNS drugs (Supplementary Table S1), at 2 or more doses. Screening this library against the behavioral profiles of an animal model of HD identified several drugs that are used clinically to treat neuropsychiatric symptoms of HD, including modafinil, bupropion, and methylphenidate (McColgan and Tabrizi, 2018), and the atypical antidepressant tianeptine, as a novel potential treatment for HD. We further showed empirical support for the DBSA method following acute and chronic treatments of Q175 mice with tianeptine.

### The Q175 model of HD

The DBSA method relies on the suitability of available animal models of disease. The Q175 model has been characterized in different laboratories and shown to recapitulate at least some of the phenotypic, molecular, and neuronal pathology of HD (Menalled et al., 2012; Smith et al., 2014; Heikkinen et al., 2020), despite having a CAG expansion rarely seen in humans. Moreover, using tools similar to those used in this study, we previously showed that phenotypic characteristics can be used to predict or “diagnose” the number of CAG repeats (Alexandrov et al., 2016). Thus, as measured behavioral features show correlation with the molecular, cellular, and network changes driven by the CAG repeat expansion, we hope that our results have a degree of translatability. The focus of this study, however, is to present a novel method to screen, in a high throughput manner, library compounds and identify those with potential therapeutic effects to relieve symptoms, at a minimum, in animal models of rare diseases. Thus, the interpretability of the phenotypic measures is not a requirement for this purpose, although the motor and grip strength abnormalities identified in HD Q175 mice (Menalled et al., 2012), are likely related to the behavioral motor features captured by SmartCube^®^ (Figure 2; Supplementary Figure S2).

### Tianeptine

As we selected the top drug predictions from DBSA, we considered possible phenotypic differences between male and female HD model mice, and therefore analyzed predictions separately for each sex. There is consistent evidence that sex-driven neurophysiological differences affect preclinical drug development outcomes (Williams and Trainor, 2018). For instance, Nestler’s group (Monteggia et al., 2007) showed that KO models of BDNF, a neurotrophin thought to play a crucial role in depression and anti-depressant responses, display significant sex differences in locomotion and depression-like behaviors, even as both sexes show reduced response to antidepressant desipramine. Hence, the weaker response predicted in female Q175 mice by our *in silico* screening compared to male (phenotypic reversal predicted at higher doses in female, see Table 1), is consistent with the above-mentioned sex-specific phenotypic differences. This differential predicted response by tianeptine in the two sexes, led us to opt for a chronic tianeptine treatment regimen in the follow-up validation experiment for female Q175 mice as opposed to the acute treatment administered to mice in the male Q175 validation experiment. As the main focus of the present study was testing the viability of high-throughput behavioral screening for drug discovery, further studies are needed to extend our results aimed at investigating the biological mechanisms involved in the potential therapeutic mechanisms of tianeptine in HD. An independent study (Zhang et al., 2018) supporting our results showed that chronic tianeptine ameliorated anxiety and depression-like behavior in animal models of HD (including the R6/1 transgenic, Q111 homozygous knock-in, and Q140 Het knock-in models). It was hypothesized that tianeptine restored impaired hippocampal synaptic plasticity through augmentation of brain-derived neurotrophic factor-tyrosine receptor kinase B signaling pathway, and subsequent normalization of AMPAR trafficking. Another proposed mechanism by which tianeptine may improve symptoms of HD is by restoring neuronal calcium signaling via store-operated Ca++ channels (Czeredys, 2020). While the underlying mechanism of tianeptine in preclinical HD models is not yet understood, several other antidepressants (e.g., SSRIs and bupropion) are already used in clinical settings (McLauchlan et al., 2022), supporting the conclusion that tianeptine may be a viable treatment in HD patients. Tianeptine is considered safe and efficacious in another neurodegenerative disease, Parkinson’s Disease (Agüera-Ortiz et al., 2021). However, as tianeptine presents an atypical pharmacology (Alamo et al., 2019), only direct tests in clinical trials can determine if these preclinical results will translate into clinical results for HD.

## Conclusion

This study is presented as an exploration and proof-of-concept of the DBSA method. We aimed at exploring the feasibility of *in silico* drug repurposing, using a combination of machine learning, high-throughput behavioral phenotyping, and animal models of disease. We tentatively showed that the method can identify drugs with therapeutic potential for neuropsychiatric symptoms of neurodegenerative disorders such as HD. Indeed, as an extension of the application of machine learning methods to drug discovery, the DBSA method exemplifies the power of combining machine learning-based *in vivo* phenotyping and *in silico* modeling. To the extent that the model of disease presents robust construct and etiological validity, this method promises to uncover further potential drugs for repurposing in other diseases with great unmet needs. Moreover, as the system is target-agnostic, potential novel polypharmacology could be discovered simply based on phenotypic reversal of the models’ feature signatures. Applying this method to a large selection of reference compounds led us to the identification of tianeptine as a putative drug for the treatment of HD patients, which we validated with additional assays, and is supported by independent studies. As a target-agnostic approach, our method can complement standard drug discovery approaches, especially for those cases in which the underlying molecular and neuronal mechanisms are not well understood. For rare diseases, DBSA presents a unique hypothesis-generating platform with actionable outcomes.

## Materials and methods

### SmartCube®

The SmartCube^®^ system is designed to measure numerous spontaneous behaviors and response to challenges in the same testing environment over a 45 min session. The proprietary hardware includes force sensors under the floor to capture fast movements such as a startle response. Several aversive stimuli are presented every 8–10 min to elicit reactive behavior, with resting periods interlaced to capture spontaneous behaviors. The aversive experimental challenges constitute a fixed standard protocol including: a change from a smooth floor to a floor constituted of small columns spaced such that the subject can place a limb in between (a misstep), but not the whole body; presentation of a small probe that delivers a mild aversive current when contacted; and a tactile air puff startling stimulus. Three orthogonally positioned video cameras provide constant 3D view. Digital videos of the subjects are processed using computer vision machine learning background segmentation followed by a standard elliptical model fit to each mouse frame image. The resulting fitted parameters describing body shape and dimensions, x-y-z coordinates of different body parts, form time series with about ½ million point each per mouse per session. A final dimensionality reduction step uses hardcoded rules and machine learning to detect and quantify over 2,000 behavioral features, such as rearing, grooming, locomotion, digging, and immobility, and their transition probabilities. Actions in the platform such as assignment of subjects to the different experimental chambers, running the standard session, starting and stopping camera recording, transferring videos, extracting behavioral features, and storing in a database occur automatically with minimal human intervention.

### Animals and experimental method

*WT mice for reference drug screening:* C57BL/6NTac male mice were received at 7 weeks of age from Taconic Farms (B6-M). Most doses were tested in groups of more than 15 (most groups were close to 20 mice). Only triazolam was tested with 9–11 mice. *HD Model DBSA Modeling Data.* Cohorts of female and male WT and Q175 het mice were received from Jax Laboratories and tested at 2 (N = 12–16), 6 (N = 9–15), and 10 months of age (N = 6–16). Such behavioral data and associated molecular biomarkers are publicly available as part of the *Mouse Htt Allelic Series Project* (Preclinical informatics: HDinHD | CHDI Foundation). *HD Model Acute Cohort:* WT male mice (C57BL6/J, Stock 000664; Jackson Laboratories; N = 15) were received at about 6 months of age. Q175 heterozygous (Het) male mice (N = 15) were generated at PsychoGenics and enrolled in the study at 6 months of age. *HD Model Chronic Cohort:* WT female mice (C57BL6/J, Stock 000664; N = 18) were ordered from Jackson Laboratories at 5 months of age. Q175 Het female mice (N = 26) were generated at PGI and enrolled at 5 months of age.

Animals received from vendors typically spent 1 week acclimatizing to the colony conditions. C57BL/6NTac mice were housed in groups of three to four in mouse Opti cages. Q175 and controls were housed in rat Opti cages with standard enrichment (play tunnels, plastic bone and enviro-dry). Mice were taken in their home cage to the experimental room area, where they remained until they were placed in the SmartCube^®^ apparatus. After the session, mice were placed back into to their home cage and returned to the colony room. Mice were body-weighted weekly during dosing. Tail samples from all het Q175 mice were collected and sent to Laragen to confirm genotypes and CAG repeats number.

### Reference drugs screening in smartCube®

To build the reference data set (Supplementary Table S1), drugs were injected at different appropriate doses using a common vehicle consisting of 5% Pharmasolve, 30% premade P3 (1:1:1 PEG200: PEG400: propylene glycol), 65% Saline. All drugs were injected *i. p.* and tested in SmartCube^®^ after a 15-min pretreatment.

### Tianeptine testing in Q175 mice

Tianeptine (Tocris) or vehicle was administered *i. p.* once in the acute group or daily for 4 weeks in the chronic group. Mice in the acute group were tested in SmartCube^®^ 15 min after injection. Animals in the chronic group were tested in SmartCube^®^ 15 min after the last injection. All drugs were dissolved in saline and injected with a dose volume of 10 mL/kg.

### DRFA

To quantify phenotypic discrimination of HD mice vs. WT, and percent recovery by acute or chronic tianeptine treatments, we used DRFA, as previously described (Alexandrov et al., 2015). This method transforms the original behavioral features into linear combinations (decorrelated features). Each decorrelated feature is a statistically independent, weighted combination of all features. This avoids overfitting and overinterpretation of certain features due to high correlation among some of the original features and reduces the dimensionality of the data without loss of relevant information. In this reduced-dimensionality feature space (DRF space), each group can be graphically represented as a *cloud*, which represents the groups’ mean (the center of the cloud), its standard deviation (outer ellipse) and the standard error of the mean (inner ellipse). DRFA quantifies the group separability as a *Discrimination Index*, which estimates the degree of overlap between the multi-dimensional probability distributions of the two groups within the DRF space. Discrimination ranges between 50% and 100%, where 50% represents no separation between the two groups and 100% represents complete separability. Once the DRF space is determined from the HD and WT group data, a test group, the treated HD mice, is projected onto the DRF space to quantify recovery or rescue of a phenotype. Recovery is calculated by first orthogonally projecting the test group onto the segment joining the centers of HD and WT clouds in the multidimensional DRF space, then the proximity of the test group mean to the WT mean is measured relative to the HD mean, along the HD-WT segment. In one extreme, if the test group overlaps (or extends further) with WT group Recovery will be 100%, if it overlaps with HD (or extends further in the opposite direction) the Recovery is 0%. We compute a *p*-value on Recovery as a parametric test based on the t-distribution comparing the test and HET groups (after projection onto the segment), with the null hypothesis being that there is no difference between the two.

### DBSA method

This method quantifies enrichment of phenotypic measures increased or decreased by a drug treatment against opposite changes observed in the animal model (target phenotype). The enrichment is computed based on an extension of GSEA (Subramanian et al., 2005), called GSEA2 (Lim et al., 2009), which tests enrichment of both increased and decreased feature sets of the target phenotype against the differential changes induced by the library compound. In GSEA2 this is accomplished in a single step, as opposed to running two separate GSEAs, one for the increased feature set and one for the decreased feature set. By screening the profile of a target phenotype against the entire database of library profiles, we obtain enrichment scores for each library compound along with a *p*-value, estimated by non-parametric statistics. Thus, we rank the library compounds by the enrichment scores to identify candidate compounds with reversed signature and select them for further validation experiments (see Supplementary Materials for details.)

### Study approval

All animal experiments used humane end points, were approved by the PsychoGenics Institutional Animal Care and Use Committee (IACUC), which meets the membership requirements of AAALAC and OLAW.

## Data Availability

The original contributions presented in the study are included in the article/Supplementary Materials, further inquiries can be directed to the corresponding author.
