# Supplementary material for: Enrichment analysis of phenotypic data for drug repurposing in rare diseases
Source: Front Pharmacol. 2023 Jul 25;14:1128562. doi: 10.3389/fphar.2023.1128562 (PMC10407094; doi:10.3389/fphar.2023.1128562)

#### Suppl. Methods

##### Drug Behavioral Signature Analysis (DBSA)

Gene expression correlative analysis uses a wide collection of genome-wide transcriptional expression data from cultured human cells or in vivo behavioral data following treatment with bioactive small molecules together with pattern-matching algorithms to discover functional connections between drugs and diseases. The underlying hypothesis for correlative analysis is that a drug effective in the treatment of a disease (or reduction of side-effects of another compound) should have opposite profile to that of the disease (or the other compound), whereas drugs with similar therapeutic indications should have similar profiles. Based on this principle, we designed a new algorithm called Drug Behavioral Signature Analysis (DBSA), which utilizes drug-induced behavioral data to predict functional connections among drugs or between drugs and diseases. In brief, this method quantifies enrichment of phenotypic measures increased or decreased by a drug treatment vs opposite changes observed in the animal model, and *p* values are computed for enrichment scores based on a non-parametric statistical analysis.

PsychoGenics Inc. created a database containing SmartCube® based phenotypic profiles of hundreds of reference compounds. We query these databases to identify compounds with potential therapeutic benefit using DBSA. This method is based on an extension of Gene Set Enrichment Analysis or GSEA (Subramanian et al., 2005), called GSEA2 (Lim et al., 2009), which tests enrichment of both increased and decreased feature sets of the target phenotype against the differential changes induced by the library compound. In GSEA2 this is accomplished in a single step, as opposed to running two separate GSEAs, one for the increased feature set and one for the decreased feature set. We query the profile of a disease model (alternatively a target compound can be used as target model), called the signature set, against the entire database of library profiles, called the list set, and identify compounds with similar or reverse profiles, depending on the underlying requirements. Briefly, it defines the signature set by comparing the disease model group to the wild type control group using t-statistics and identifies features which are significantly different (with *p*-value < 0.5) and partition these features into increased (S+) and decreased (S-) feature sets. Next, it compares the SmartCube® data of a library compound to the vehicle group using t-statistics and ranks all features by t score, from the most increased to the most decreased. The method then uses the Kolmogorov-Smirnov statistics test to assess whether S+ and S- are statistically enriched in the two extremes of the ranked list features. This enrichment is quantified as Enrichment Score (ES). For a randomly distributed S+ and S-, ES will be relatively small, but if it is concentrated at the top and/or bottom of the list then ES will be correspondingly high. Samples labels in the disease model and wild type groups are randomly shuffled 1000 times to estimate the ES null distribution. This permutation preserves correlations between different features and thus generates a biologically relevant null distribution. Statistical significance of the ES is computed by comparing the observed ES to the null distribution.

This analysis has three different outcomes: a) ‘similar’ indicating a profile similar to that of the disease model; b) ‘reverse’ indicating a profile opposite to that of the disease model, thus suggesting the therapeutic potential on the disease; and c) ‘random’ indicating no similarity. In the final output two statistics that measure the level of enrichment of differential features shared by the disease model and the library compound are computed: Normalized Enrichment Score (NES) and the Odds Ratio (OR). The NES is calculated as by rescaling the ES by the mean of randomized enrichment scores (mean is calculated only for the randomizing yielding the same sign as ES) (Subramanian et al., 2005). OR is defined as the ratio between the odds of the leading edge (LE) features belonging to signature set and the odds of the LE features not belonging to the signature set, where the LE is defined by the top segment of the ranked list of features up to the feature with the maximum running ES for positive enrichment scores and minimum ES for negative enrichment score.

**Suppl. References**

Lim, W.K., Lyashenko, E., and Califano, A. (2009). Master regulators used as breast cancer metastasis classifier. *Pac Symp Biocomput***,** 504-515.

Subramanian, A., Tamayo, P., Mootha, V.K., Mukherjee, S., Ebert, B.L., Gillette, M.A., et al. (2005). Gene set enrichment analysis: a knowledge-based approach for interpreting genome-wide expression profiles. *Proc Natl Acad Sci U S A* 102(43)**,** 15545-15550. doi: 10.1073/pnas.0506580102.

#### Supplementary Tables

| **Table 1 (Suppl.):** Reference drugs tested and their ranges | | | |
| --- | --- | --- | --- |
| Drug | Dose range (mg/kg) | Drug | Dose range (mg/kg) |
| alprazolam | 0.1-1 | methylphenidate | 2.5-10 |
| amitriptyline | 2.5-10 | milnacipran | 30-45 |
| D-amphetamine | 0.5-4 | mirtazapine | 8-64 |
| aripiprazole | 0.25-2 | moclobemide | 3-80 |
| acetylsalicylic acid | 200-300 | modafinil | 7.5-120 |
| atomoxetine | 2-4 | morphine | 0.6-3 |
| bupropion | 8-32 | MPEP | 20-40 |
| buspirone | 0.5-12 | naproxen | 80-100 |
| caffeine | 5-10 | nefazodone | 5-40 |
| chlordiazepoxide | 4-64 | Nicotine | 0.25-1 |
| chlorpromazine | 1-6 | nortriptyline | 10-20 |
| citalopram | 1.5-60 | olanzapine | 0.1-2 |
| cocaine | 2.5-10 | oxycodone | 0.25-0.5 |
| codeine | 5-7.5 | oxazepam | 0.8-10 |
| clorazepate | 5-10 | paroxetine | 16-32 |
| clonazepam | 0.025-5 | phencyclidine | 5-10 |
| clozapine | 1-6 | physostigmine | 0.3-0.6 |
| diflunisal | 60-80 | perphenazine | 0.25-3 |
| diphenhydramine | 10-45 | PTZ | 25-75 |
| desipramine | 5-60 | quetiapine | 1-12 |
| DOI | 1-3 | reboxetine | 15-30 |
| donepezil | 0.9-1.8 | risperidone | 0.04-0.75 |
| amitifadine | 10-20 | rolipram | 0.5-20 |
| duloxetine | 15-45 | sertraline | 5-80 |
| diazepam | 0.25-8 | tacrine | 2-4 |
| escitalopram | 3-60 | temazepam | 0.5-5 |
| flesinoxan | 0.15-0.6 | thioperamide | 45-90 |
| fluoxetine | 10-40 | tiagabine | 1-20 |
| fluvoxamine | 16-64 | tianeptine | 10-60 |
| gabapentin | 25-100 | tramadol | 10-100 |
| haloperidol | 0.1-1 | trazodone | 10-20 |
| hydroxybupropion | 16-32 | triazolam | 0.5-1 |
| imipramine | 5-60 | valproate | 20-400 |
| ipsapirone | 0.5-9 | venlafaxine | 10-80 |
| lamotrigine | 10-20 | yohimbine | 2-20 |
| lithium | 200-600 | zaleplon | 0.5-1 |
| lorazepam | 0.1-0.4 | ziprasidone | 0.1- 5 |
| MCPP | 1-10 | zolpidem | 0.5-1 |
| memantine | 10-20 |  |  |

#### Supplementary Figures

**Figure 1 (Suppl.)**. DRFA analysis of male and female HD mice at 2, 6, 10 months versus their corresponding WT control. In this reduced-dimensionality feature space (DRF space – See methods), each group is represented as “clouds” showing the mean (center), standard deviation (outer ellipse) and the standard error of the mean (inner ellipse). The Discrimination Index is calculated for each pairwise comparison. P-values are shown for each comparison.


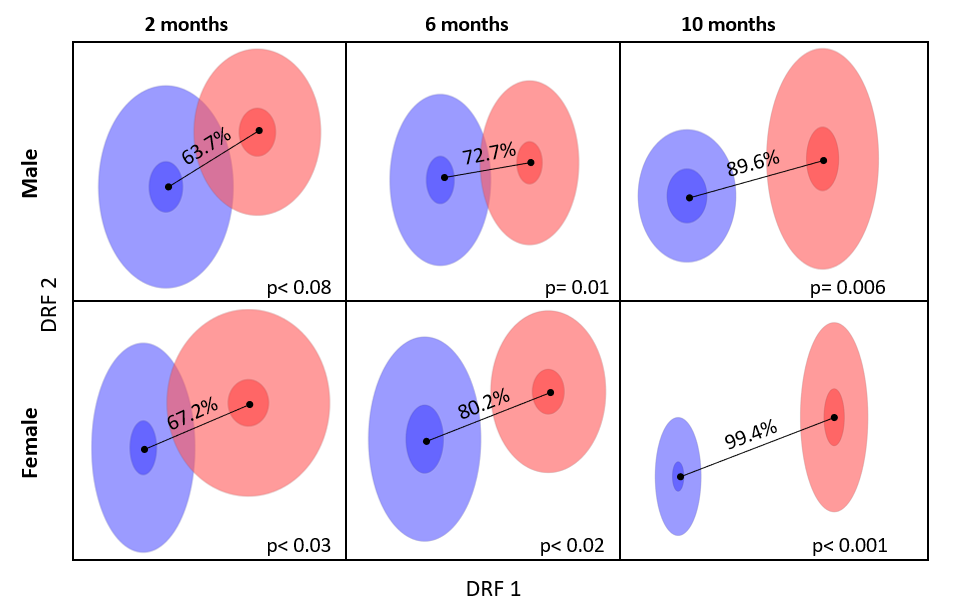


**Figure 2 (Suppl.)**. Top features derived from the DBSA analysis of male and female HD mice at 2, 6, 10 months versus their corresponding WT control (see Figure 1, Suppl.). The SmartCube® system captures more than 2000 features, here we depict the top 20 most decreased and increased features, as compared to the WT group. Features with a (-) symbol represent latencies (e.g. latency to rear), whereas the remaining represent counts or times engaged in particular behaviors (e.g. time rearing). The same behaviors are measured repeatedly over an experimental session, and in different ways.


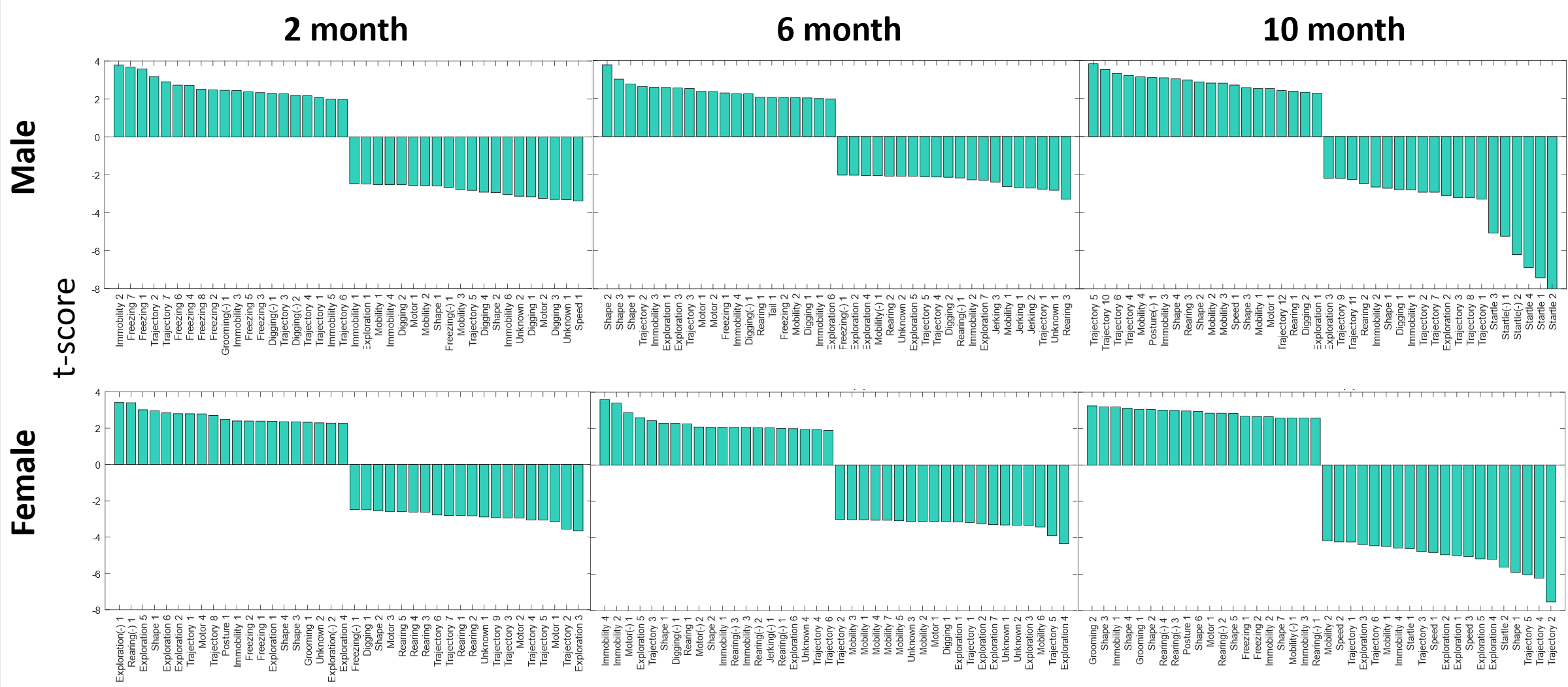


**Figure 3 (Suppl.)**. Top features derived from the DBSA phenotypic analysis of male (top) and female (bottom) HD mice at 6 months of age are plotted as t-score of HD vs WT (blue bars). Overlayed yellow bars represent the t-score of tianeptine vs vehicle an independent cohort of HD mice treated with tianeptine (acute 20 mg/kg *i.p.,* in males; chronic 20mg/kg *i.p.*, in females). This representation highlights the feature-by-feature recovery, where rescued features are those for which blue and yellow bars have opposite direction.


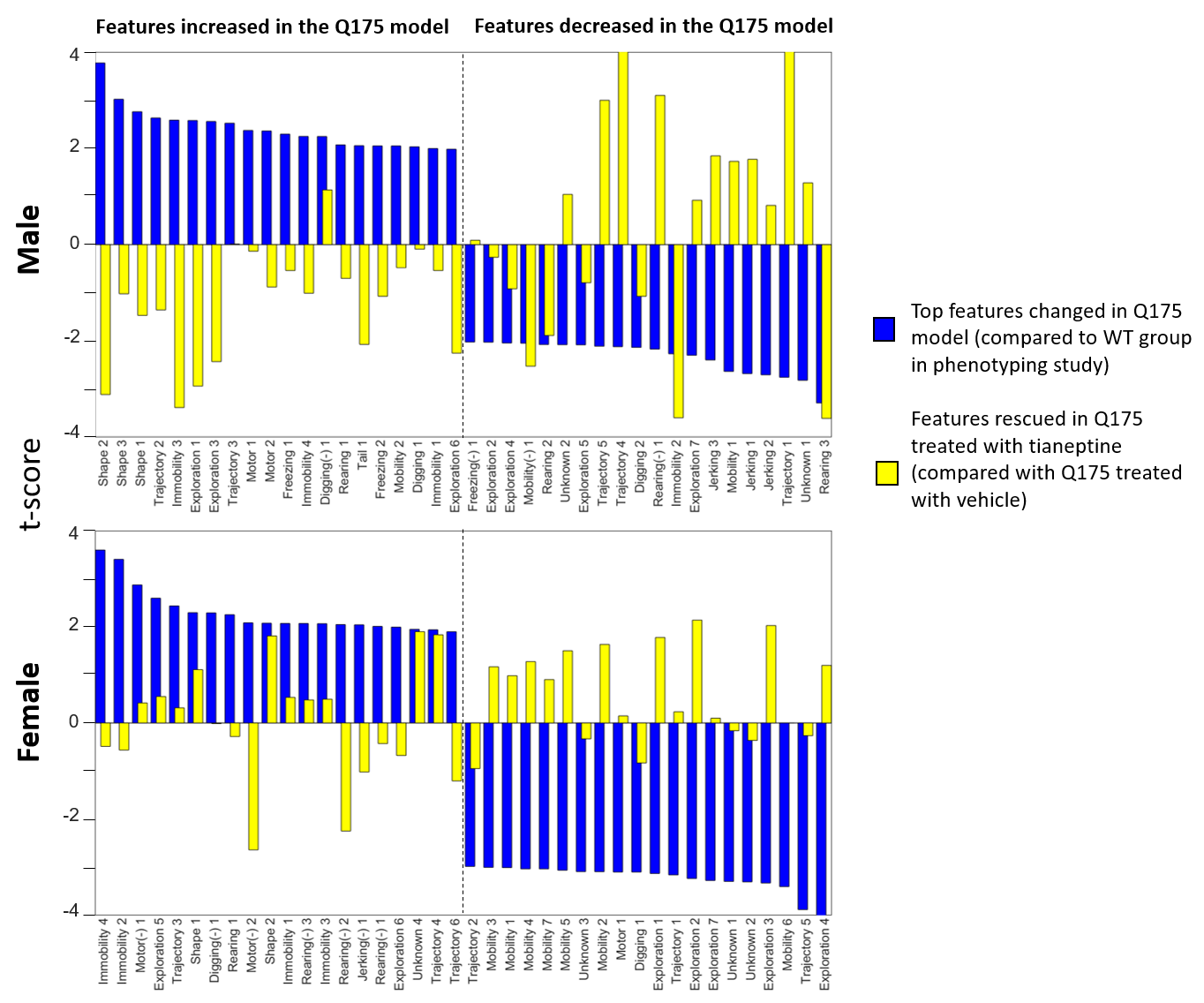


**Figure 4 (Suppl.)**. The difference between the tianeptine treated mice vs vehicle controls (as part of the large drug profile collection), were quantified with a t-score for each feature. Similarly, t-scores were derived from the comparison of the Q175 mice treated with tianeptine or vehicle. In both cases treatments were administered *i.p.*, acutely in male mice. The scattergram depicts the Q175-tianeptine t-scores as a function of the WT-tianeptine t-scores.


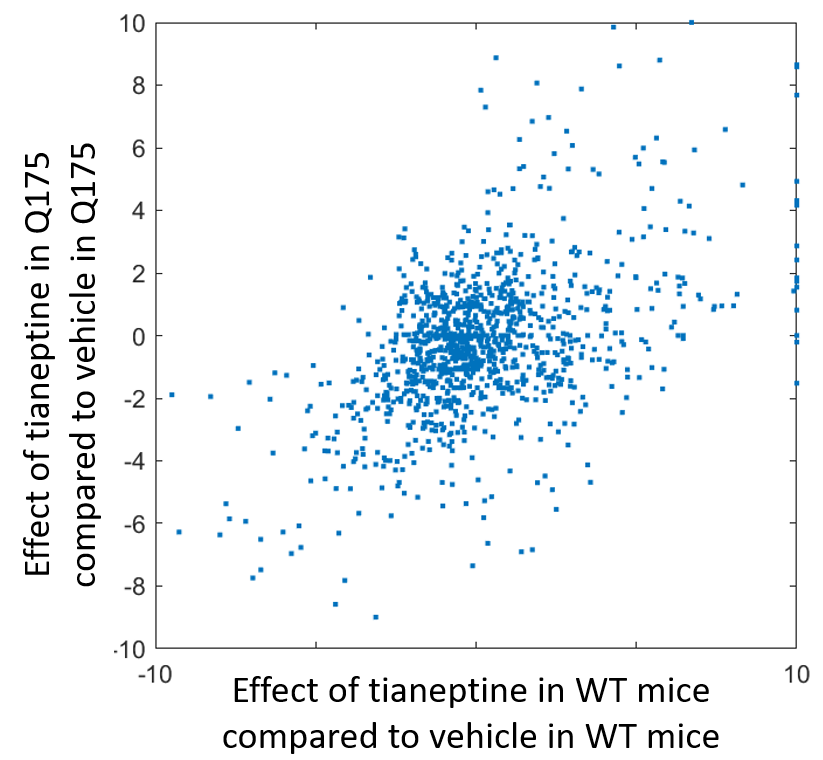

Supplement: Supplementary file 1 [file Table1.DOCX]
